# Circulating microRNAs as potential cancer biomarkers: the advantage and disadvantage

**DOI:** 10.1186/s13148-018-0492-1

**Published:** 2018-04-23

**Authors:** Hao Wang, Ran Peng, Junjie Wang, Zelian Qin, Lixiang Xue

**Affiliations:** 1 Medical Research Center, Peking University Third Hospital, Beijing, China; 2 Department of Radiation Oncology, Peking University Third Hospital, Beijing, China

**Keywords:** Cancer, Circulating microRNA, Biomarker, Diagnosis, Prognosis

## Abstract

MicroRNAs are endogenous single-stranded non-coding small RNA molecules that can be secreted into the circulation and exist stably. They usually exhibit aberrant expression under different physiological and pathological conditions. Recently, differentially expressed circulating microRNAs were focused on as potential biomarkers for cancer screening. We herein review the role of circulating microRNAs for cancer diagnosis, tumor subtype classification, chemo- or radio-resistance monitoring, and outcome prognosis. Moreover, circulating microRNAs still have several issues hindering their reliability for the practical clinical application. Future studies need to elucidate further potential application of circulating microRNAs as specific and sensitive markers for clinical diagnosis or prognosis in cancers.

## Background

Cancer is one of the leading causes of death worldwide. In recent years, some significant improvements have been made in tumor diagnosis and treatment. However, early detection is still critical for improving outcomes and reducing recurrence and mortality of cancer patients. The absence of obvious symptoms and insufficiently sensitive biomarkers in early stages of carcinoma limits early diagnosis. Biopsy and imaging examination as golden standards greatly improve the detection rate, but their applications are limited by their own invasive or radiation-related characteristics, respectively. In addition, traditional tumor diagnostic markers like carcinoembryonic antigen (CEA) and CA199 usually exhibit low sensitivity. Therefore, it is urgent to identify novel, more sensitive, and easy-to-detect biomarkers which can be used in diagnosis and prognosis of cancers.

Novel methods are currently under development for cancer detection, including those based on the detection of microRNAs (miRNAs). MicroRNAs are endogenous, single-stranded, non-coding small RNA with length of ~ 22 nucleotide (nt). The first miRNA was discovered in 1993 in *Caenorhabditis elegans* which participated in embryo development [1]. In the next two decades, miRNAs were found in plants, animals, protists, and viruses but not in bacteria. These small RNA molecules function as antisense RNA to negatively regulate their target genes at the post-transcription level. Most miRNAs have only modest effects on the translation of their target genes, but they constitute highly complex networks with their targets and downstream effectors [2]. A single gene is simultaneously targeted by multiple miRNAs, while each miRNA is able to target numerous genes via similar seed sequence. It was reported that miRNAs regulated more than 30% of the human genome and are involved in almost all fundamental cell processes [3, 4]. A group of target genes may function through a common signaling pathway and accordingly facilitate similar cell behaviors, such as proliferation, apoptosis, differentiation, migration, invasion, metabolism, and stress response.

The expression patterns of miRNAs are usually altered in different development stages and various pathology conditions like senescence, cardiovascular diseases, and cancers [2, 5, 6]. Dysregulations of miRNAs were often observed in different kinds of cancers due to dysfunction of the miRNA biogenesis process, transcription of miRNA-encoding genes, and regulator of mature miRNAs like circular-RNA [2]. Some of these altered miRNAs were significantly overexpressed and regarded as oncogenes or “oncomiRs” which accelerate tumor occurrence, development, and metastasis. Meanwhile, those that decreased in cancer patients were considered tumor suppressors [2, 6]. The differential expression of miRNAs can be detected by polymerase chain reaction (PCR), Northern blotting, microarray, and deep sequencing and have potential for clinical applications [7, 8].

miRNAs in different cell types can be secreted into the extracellular space and then transported to the circulating body fluid like peripheral blood. It was reported that 10% of the known human miRNAs could be detected in plasma. About 30% of them were mirtrons, rare miRNAs originated from short-chain, hairpin-structured introns of mRNA and through a special biogenesis pathway [9–11]. These miRNAs are detectable in plasma or serum in a remarkably stable form, encapsulated into the extracellular vesicles or bound with special lipid proteins, thus being resistant to RNase digestions [12–15]. Therefore, these small molecules are capable to be ideal candidates to serve as biomarkers for cancer detection by liquid biopsies. Besides peripheral blood, various body fluids, including saliva, cerebrospinal fluid, ascites, urine, breast milk, and semen, allow for miRNA detection [16].

This review mainly discussed the potential application of circulating miRNAs as clinical cancer biomarkers. We herein are more focused on those cancers with higher incidence rates and mortalities, more difficult detection in early stages, and heavier burdens on people and society, like cancers of the lung, liver, colorectal, stomach, and breast.

## Potential clinical application of circulating miRNAs

**Fig. 1:**
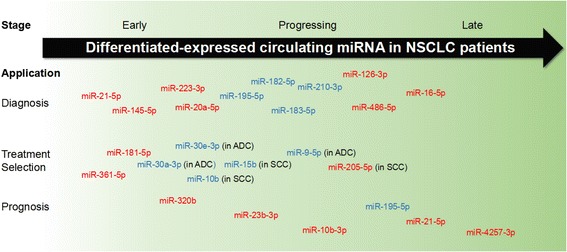
The potential clinical applications of circulating miRNAs as tumor biomarkers for NSCLC. Diverse circulating miRNAs functioned at various aspects of clinical screening. In NSCLC patient for example, some differentially expressed miRNAs facilitated cancer diagnosis and prognosis while others contributed to treatment strategy selection by distinguishing tumor subtypes, monitoring tumor progression, or predicting the drug resistance. The expression levels of these cell-free miRNAs could be significantly differentiated at early stage, during the tumor progression, or until the late stage. Red color indicated increased expressed miRNAs in peripheral blood of NSCLC patients, while blue color indicated decreased ones

The differential expression of circulating miRNAs exhibited promising potential for cancer screening without additional injury for patients. The abnormal levels of distinct miRNAs could be observed at an early stage, during progression, and after metastasis of cancers. Thus, these small RNA molecules may function as favorable clinical biomarkers for distinguishing tumors, treatment strategy selection, and outcomes. In non-small cell lung cancers (NSCLC) patients, for example, a large group of miRNAs have been identified to be differentially expressed in different stages of disease and to contribute to the diagnosis, treatment determination, and prognosis (Fig. 1).

### Circulating miRNAs as biomarkers for early diagnosis

A growing number of circulating miRNAs was reported to be dysregulated in the early stage of cancers. The altered expression may be observed before the obvious clinical symptoms or clear biopsy and image examination evidence. Plasma miR-21-5p, miR-20a-5p, miR-141-3p, miR-145-5p, miR-155-5p, and miR-223-3p significantly increased for NSCLC patients at stages I and II [17–19]. Serum miR-126-3p, miR-182-5p, miR-183-5p, and miR-210-3p were also found to possess early detective value for NSCLC patients, exhibiting similar sensitivity and specificity with traditional tumor marker CEA [20]. Two miRNA precursors, pri-miR-944 and pri-miR-3662, were also capable of distinguishing NSCLC at stages I–IIIA [21]. Significantly decreased levels of miR-125a-3p were observed in plasma exosomes of colon cancer patients [22], as well as increased levels of miR-23a-3p, miR-27a-3p, miR-142-5p, and miR-376c-3p in serum [23]. A group of miRNA, including miR-642b-3p, miR-1202-5p, miR-1207-5p, miR-1225-5p, miR-4270-5p, and miR-4281-3p, was upregulated in plasma of breast cancer patients with stage I [24]. Serum miR-1825-3p was specifically downregulated in glioma at early stage, and its level was correlated with tumor progression and poor prognosis [25]. These evidences suggest that the circulating miRNA detection might be introduced for early-stage cancer screening.

### Circulating miRNAs as diagnosis biomarkers to distinguish different subtypes of cancer

Cancers can be divided into different subtypes by tissue origination or pathological mechanisms. For example, NSCLCs include two major pathologic subtypes, adenocarcinoma (ADC) and squamous cell carcinoma (SCC) [26]. Breast cancers are well known as heterogeneous diseases, which can be sub-classified by the presence of estrogen receptor (ER), progesterone receptor (PR), and HER2/neu receptor [27, 28]. The sub-classification of specific tumors is valuable for determining tumor mechanisms and making therapeutic decisions. Of note, certain differentially expressed miRNAs in parallel with the different subtypes draw more and more attention recently. They could be optimal to determine tumor subtype and pathology, contributing to the selection for a more efficient therapeutic approach.

Taking NSCLC as an example, the accurate sub-classification into ADC and SCC is important for deciding treatment methods. Expression patterns of several miRNAs were different not only between the plasma of NSCLC patients and healthy individuals, but also between ADC and SCC patients. Levels of miR-16-5p and miR-486-5p were elevated in ADC and SCC cases compared to those of healthy ones. miR-9-5p expression was stable between overall NSCLC patients and healthy controls, but exhibited significant declination in ADC patients instead of SCC ones. Another plasma miRNA, miR-205-5p, was upregulated only in SCC patients [29]. Additionally, Jin et al. found several ADC- and SCC-specific differentially expressed miRNAs by RNA sequencing [30]. miR-181-5p, miR-361-5p, and miR-320b were significantly elevated in plasma exosomes of NSCLC patients. The levels of miR-181-5p and miR-361-5p were increased by more than 10 times in ADC patients than SCC patients, and miR-320b in SCC samples increased by over 10 times than in ADC ones. miR-30a-3p and miR-30e-3p were specifically downregulated in ADC patients, while miR-10b-5p and miR-15b-5p were decreased in SCC patients. Therefore, investigators suggest that these miRNA panels may be not only applicable in NSCLC diagnosis, but also helpful to subtype discrimination. Interestingly, cell-free miRNA precursors were also found to have diagnostic potential. Pri-miR-944 was suggested to distinguish SCC from ADC, while pri-miR-3662 distinguished ADC from SCC [21]. In addition, the mature forms of these two miRNAs were also revealed to have the potential in indicating the diagnostic accuracy for operable SCC and ADC, respectively [31].

The subtyping of heterogeneous breast cancer is also of great importance for clinical therapy. The positively expressed ER, PR, or HER2/neu in tumors could be directed as therapeutic targets. On the contrary, patients bearing triple-negative breast cancer (TNBC), which expressed none of these receptors, were usually treated with traditional chemotherapy or radiotherapy [27]. TNBC was associated with higher stage at diagnosis and poorer prognosis. Therefore, biomarkers of specific breast cancer subtypes have also been focused on, especially for TNBC from the miRNA point of view. Shin et al. [32] observed the declined levels of miR-16-5p, miR-21-5p, and miR-199a-5p in plasma and tumor tissues of TNBC patients compared with both non-TNBC and healthy individuals, as well as the elevated levels of miR-92a-3p and miR-342-3p. Among them, miR-199a-5p exhibited the highest value to distinguished TNBC from non-TNBC. This small molecule was validated to be downregulated since the stage I of tumor and to decrease with tumor progression [32]. Meanwhile, exosomal levels of miR-373-3p were increased in TNBC but not luminal carcinomas and higher in ER/PR-negative tumors than receptor-positive tumors [33]. Interestingly, serum levels of miR-373-3p showed no significant difference between TNBC and luminal carcinomas.

In other cancers, different tumor subtypes may also be distinguished by circulating miRNAs. Exosomal levels of miR-101-3p and miR-483-5p in plasma of adrenocortical cancer were significantly higher than those of adrenocortical adenomas, which could be adopted to preoperative diagnosis of adrenocortical malignancy [34]. On the other hand, papillary and follicular thyroid cancer might be distinguished by the expression of exosomal miR-21-5p, miR-31-5p, and miR-181a-5p in combination [35].

### Circulating miRNAs contribute to monitor tumor metastasis

The occurrence of tumor metastasis leads to a significant impairment of curative effect, resulting to the poor survival rate and high risk of recurrence. There are currently no reliable biomarkers for predicting metastatic spread to different sites. According to the characteristics of relative tissue specificity of miRNAs, the candidates for this purpose become favorable since more and more circulating miRNAs were found to be associated with clinical tumor stage and/or metastasis. In osteosarcoma patients, miR-497-5p was significantly downregulated in primary tumor tissues, metastatic tissues, and serum compared to healthy controls [36–41]. This small molecule targeted multiple genes like IGF-1R [38], VEGFA [39], AMOT [40], and P21 [42] to inhibit osteosarcoma cell proliferation, migration, and invasion and enhance apoptosis. Furthermore, the declined miR-497-5p expression was associated with clinical stage, distant metastasis, and promoted cisplatin resistance [36, 39]. Elevated levels of miR-205-5p in plasma were correlated with tumor stage and pathological grade in patients with bladder cancer [43]. miR-148a-3p expression was reduced in plasma of ovarian cancer patients and correlated with histopathologic grade and lymph node metastasis [44]. Plasma levels of miR-520g were higher in breast cancer patients with low differentiation degree grade, mammary gland invasion, and lymph node metastasis [45]. Additionally, both miR-106a-5p and miR-196a/b-3p were upregulated in gastric cancer patients, and their levels were associated with TNM stage and metastatic potential [46, 47]. In the future, further clarifying the miRNAs from the original tumor sites or the targeted metastatic tissue/organ might be helpful to predict the upcoming metastasis event.

### Circulating miRNAs predicted the sensitivity of tumor to clinical treatment

Chemotherapy and radiotherapy are important approaches for tumor treatment, which are often in combination with surgical operation. However, some types of tumor cells may gain resistance after long-time treatment due to the heterogeneity of tumors. As a result, parts of patients would have worse outcomes upon a certain treatment. Investigators expect to find accurate biomarkers to detect or predict the resistance of different cancers, in order to select superior treatment strategies.

In pancreatic ductal adenocarcinoma (PDAC), a main subtype of pancreatic cancer, miR-155-5p was upregulated in tumor tissues and plasma, and the expressions in tissues were associated with tumor stage and poor prognosis [48–50]. Besides, long-term administration of gemcitabine in tumor cells further overexpressed miR-155-5p which was released into exosomes to induce gemcitabine resistance via anti-apoptotic activity. Therefore, higher miR-155-5p expression showed chemoresistance and poor prognosis for PDAC patients receiving gemcitabine treatment [51]. Levels of miR-1914-3p and miR-1915-3p in plasma from chemo-resistant colorectal cancer (CRC) patients were decreased compared to those from responders. These two miRNAs were demonstrated to facilitate cell resistance to 5-Fu and oxaliplatin by NFIX in vitro [52]. Aberrant reduction of miR-497-5p in plasma of osteosarcoma patients implied the poor response to chemotherapy [36]. Furthermore, lower miR-146-5p levels in serum exosomes were associated with the cisplatin resistance and shorter progression-free survival (PFS) for NSCLC patients [53]. These putative resistant miRNAs may be favorable for monitoring the resistant and tolerance of treatment and for selection of clinical therapeutic approach. In line with these findings, targeting those miRNAs and their downstream targets might become the novel strategy to rescue the resistance against chemotherapy or radiotherapy.

### Circulating miRNAs as prognostic biomarkers of cancers

Accumulating evidences suggested that circulating levels of miRNAs may be associated with the outcomes. The expressions of miR-222-3p in serum exosomes were associated with poor outcomes of NSCLC patients [54]. After internalized via caveolin- and lipid raft-mediated endocytosis, miR-222-3p promoted the proliferation, chemo-resistance, migration, and invasion of recipient cells. Elevated levels of miR-23b-3p, miR-10b-3p, and miR-21-5p in exosomes were associated with poor overall survival (OS) of NSCLC patients [55]. Additionally, higher levels of miR-21-5p and miR-4257-3p existed in exosomes of recurrent NSCLC patients compared with those without recurrent and the healthy controls [56]. The level of these two exosomal miRNAs was associated with disease-free survival (DFS), while miR-4257-3p alone was also associated with node metastasis and TNM stage. On the other hand, miR-21-5p was also upregulated in the circulating exosomes, primary tumor tissues, and liver metastasis tissues [57]. Exosomal level of miR-21-5p was associated with liver metastasis, TNM stage, and poor prognosis, including shorter OS and DFS.

Besides the tumor type mentioned above, circulating miRNAs also showed tight correlation with prognosis in other types of tumors. The levels of let-7b and miR-18a-5p were significantly associated with DFS and OS in multiple myeloma patients [58]. miR-4772-3p levels were negatively associated with the risk of recurrence and death in serum exosomes derived from stage II and stage III colon cancer patients [59]. Higher levels of plasma miR-148a were associated with longer OS in ovarian cancer patients [44, 60]. Lower plasma levels of miR-185-5p were correlated with poor survival in glioma patients [61]. These findings above suggest the promise of applying circulating miRNAs as biomarkers for early prediction upon certain treatment and improvement of the outcomes in most types of cancer.

## The advantage of circulating miRNAs as diagnosis and prognosis biomarkers

Traditional cancer markers are mainly produced by tumor tissues or normal embryo tissues, while absence or in tiny amount in tissue and blood of healthy adults. The most validated traditional cancer markers include alpha-fetoprotein (AFP), carcinoembryonic antigen (CEA), and carbohydrate antigen (CA) [62, 63]. They are generally broad-spectrum biomarkers for diagnosis of various types of cancers. CEA, CA199, and CA125 were generally accepted to be validated to have positive predictive value as circulating biomarkers for different cancers. Other screening strategies include mammography for breast cancer [64, 65], colonoscopy for CRC [66], and prostate-specific antigen (PSA) for prostate cancer [67]. However, still missing are more effective, accurate, specific, and sensitive screening biomarkers to fulfill the detective and predictive functions in the care of cancer patients. Circulating miRNAs have the particular advantage as a potential clinical application.

### Circulating miRNAs are non-invasive biomarkers

Circulating miRNAs are easy to obtain without severe damage. Besides, a great number of potential effective miRNA biomarkers are stable in healthy people. Their expression levels may not be obviously affected by age, gender, body mass index (BMI), smoking status, or other basic characteristics when evaluating pathogenic potential. Hence, the altered expression pattern might be introduced to routine examination for monitoring and early diagnosis of cancers.

Although cell-free miRNAs from plasma and serum are the most common circulating miRNA biomarkers, other body fluid samples like urine and saliva are also applicable as the resource of circulating miRNAs. miR-186-5p was overexpressed in not only tumor tissues and blood, but also urine from bladder cancer patients [68]. Several miRNAs including miR-210-3p were upregulated in urine from transitional cell carcinoma patients and capable to facilitate cancer diagnosis [69]. All members of let-7 families were significantly elevated in urine from clear-cell renal cell cancer (ccRCC) patients [70], while a combination of urinary exosomal miR-34b-5p, miR-126-3p, and miR-449a-3p could be favorable for ccRCC diagnosis [71]. A panel of four breast cancer-related miRNAs (miR-21-5p, miR-125b-5p, miR-155-5p, and miR-451-5p) was also differentially expressed in urine samples of breast cancer patients and exhibited their diagnostic value [72]. Elevated levels of miR-143-3p and miR-30e-5p in urine samples of PDAC patients shown their potential as diagnostic biomarkers in the early stage [73]. Therefore, these easily accessible cell-free small molecules have been widely applied for clinical use.

### Circulating miRNAs may be used for screening tumors with higher sensitivity

Up to now, polymerase chain reaction (PCR) is still the major examination technology for circulating miRNA. Amplification is a critical step and characteristic of all kinds of PCR, which magnifies the initial difference between samples, even if the difference is quite small. Therefore, the current detection method makes circulating miRNAs more sensitive biomarkers. Monitoring of the aberrant expression can be easier and earlier compared with biopsy and/or image examination which reflects the actual size without amplification, although the latter are regarded as the golden standard so far.

### The dynamic expression pattern of circulating miRNAs may be associated with the progression of tumors

The generation of miRNAs is dynamic and prompt upon the internal or external stimuli. This feature endows miRNAs the ability to observe the whole time course changes in real time and dynamic manner from tumorigenesis throughout the following progression. miR-195-5p was reported to inhibit the proliferation, migration, and invasion of NSCLC cells via multiple targets [74–77], and the lower miR-195-5p levels in plasma were showed to be associated with lymph node metastasis and advanced clinical stage [78]. Serum miR-373-3p was downregulated in pancreatic cancer patients, and miR-373-3p level was negatively correlated with TNM stage, lymph node metastasis, and distant metastasis [79]. In periampullary carcinoma, miR-192-5p levels were increased and correlated with tumor stage and aggressiveness [80]. A panel of miRNAs including miR-34a-5p, miR-34b-5p, and miR-34c-5p was significantly downregulated in TNBC patients. Among these miRNAs, miR-34a-5p expression was positively correlated with lymph node metastasis together with miR-34b-5p and correlated with tumor grade and distant metastasis together with miR-34c-5p [81]. These observations revealed the possibility of circulating miRNAs for evaluating the stage and progression of tumors. The whole dynamic expression pattern of miRNAs could depict the development landscape of cancer during the entire progression.

## The disadvantage and limitation of circulating miRNAs as diagnosis and prognosis biomarkers

### The diversified origin of circulating miRNAs influences the effectiveness partially

Most of potential miRNA biomarkers ubiquitously exist in both healthy individuals and cancer patients. The differences in their expression levels between healthy people and patients are usually quite tiny. So the way of sampling cannot be ignored to distinguish cancers from healthy state or other benign injury accurately.

Currently circulating miRNAs are obtained from venous plasma or serum. miRNA profiles in venous and arterial plasma were largely similar, so the levels of most miRNAs have no significant difference between vein and artery. Five elevated miRNAs (miR-20b-3p, miR-28-3p, miR-192-5p, miR-223-3p, and miR-296-5p) were identified from serum of esophageal squamous cell carcinoma (ESCC) patients [82]. The investigators compared their content in venous and arterial serum and found no statistic difference. However, the use of venous miRNAs for cancer detection was still challenged in some cases. Ten arterial highly expressed miRNAs and fourteen venous highly expressed miRNAs were identified in plasma samples of healthy male rats [83]. The miRNA profiles in arterial plasma showed higher correlation with that in tissue. Studies with human samples had similar observations. Levels of upregulated miRNAs (miR-10-3p, miR-21-5p, miR-409-3p, and miR-425-5p) were even higher in arterial plasma compared with venous plasma from lung adenocarcinoma patients [84]. Levels of let-7g-5p, miR-15b-5p, miR-155-5p, and miR-328-5p were found to be significantly higher in mesenteric vein than in peripheral vein and tumor tissue from colon cancer patients, suggesting that tumor drain vein contained more complete biomarkers origin from tumor tissues than peripheral vein [85]. Thus, the blood sampling methods should be carefully considered for some specific miRNA biomarkers.

Exosomal miRNAs in peripheral blood have also drawn more and more attention in the aspect of biomarkers. It is known that most of the miRNAs in blood were packaged in extracellular vesicles like microvesicles and exosomes. Several miRNAs were reported to differently express in plasma, serum, and peripheral blood exosomes. For example, miR-181b-5p and miR-21-5p were enriched in the exosomes of lung cancer patients instead of healthy individuals [86]. Plasma levels of miR-19b-3p, miR-21-5p, miR-221-3p, miR-409-3p, miR-425-5p, and miR-584-5p were elevated in lung adenocarcinoma patients, but only miR-19-3p, miR-21-5p, and miR-221-3p were found to be upregulated in plasma exosomes [84]. Similarly, a panel of five serum miRNAs overexpressed in ESCC patients, including miR-20b-5p, miR-28-3p, miR-192-5p, miR-223-3p, and miR-296-5p. Only miR-296-5p was found to be upregulated in ESCC serum exosomes [82]. Likewise, miR-132-3p and miR-185-5p were overexpressed in gastric cancer patients’ serum instead of exosomes [87]. miR-101-3p was elevated both in serum and exosomes from breast cancer patient, but expression levels of miR-372-3p and miR-373-3p increased in exosome and serum, respectively [33]. These observations implied the importance of selecting a proper sampling method for certain circulating miRNAs.

### Single miRNA molecule has limitations in both sensitivity and specificity

High sensitivity and specification are the fundamental demands and the most important evaluation criteria for circulating miRNAs as diagnostic or prognostic biomarkers for clinical application. Single miRNA molecules could hardly meet the criteria for many candidate miRNA biomarkers because their levels in patients and healthy controls were overlapped. This observation suggested that the level of an applicable miRNA biomarker should possess high individual difference, which increased the possibility of false negative or positive diagnosis.

On the other hand, many cell-free miRNAs showed altered expression patterns in various types of cancers instead of a certain cancer type. miR-21-5p, miR-155-5p, and miR-210-3p are good examples, as all of them are involved in cancers like NSCLC [17–20, 88], breast cancer [89–92], and colorectal cancer [57, 93, 94]. Additionally, there was similar expression between benign injury and malign tumor. miR-21-5p levels in plasma significantly increased in CRC patients, but this candidate biomarker could not distinguish the carcinoma and benign polyps [95]. Therefore, there should be a strict process of screening from bench to bedside.

**Fig. 2:**
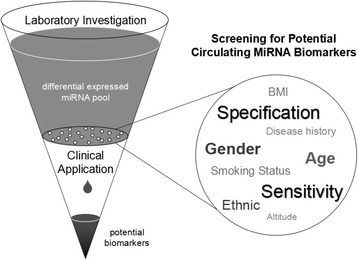
Screening for potential circulating miRNA biomarkers for clinical applications. A great number of differentially expressed miRNAs were identified in laboratory investigations to constitute the candidate pool. In the further screening, candidates which had no correlation with individual characteristics were retained, as well as those with high specificity and sensitivity in order to distinguish cancer patients from healthy people accurately. They were considered as potential clinical biomarkers for large-scale validation

Therefore, a larger sample size was essential to obtain the basal line of candidate of interest and decide whether it could clearly separate the health and disease status. Only those who have high sensitivity and specificity in people with different characteristics have the potential for clinical application (Fig. 2). In addition, more advanced technologies like digital PCR were developed in last years. These advanced methods could be further appropriate to overcome these difficulties.

Contribution of miRNAs in different cancers is complicated. A certain miRNA can be oncomiR in this kind of tumor and suppressor in another. Circulating miR-21-5p was reported to be upregulated in patients of NSCLC [17–19, 55, 56, 72], liver cancer [57], and gastric cancer [96, 97], but downregulated in patients suffered from breast cancer [32, 72]. The levels of miR-195 in peripheral blood were lower in patients with hepatocellular carcinoma [98] and cervical cancer [99]. On the contrary, its increased plasma levels were observed in osteosarcoma patients [100] and associated with poor prognosis of head and neck cancer patients [101]. It was of great importance for careful assessment when discussing the clinical application value for the certain miRNA molecules in a certain tumor.

Recently, more and more investigators turned to incorporate several miRNAs to improve the diagnostic effect or combine miRNAs with traditional biomarkers like CEA. Several panels of miRNAs were already introduced to ESCC detection [102]. Plasma levels of miRNA pair miR-19b-3b and miR-297-5p were found to be diagnostically significant for prostate cancer [103]. A combination of exosomal miR-126-3p, miR-449a-5p, and miR-34b-5p was adopted to the diagnosis of ccRCC, and combination of miR-126-3p and miR-34b-5p could identify carcinoma and benign injury [71]. It appears that the combination of different miRNAs or miRNAs with other clinical indicators will be the tendency for precise cancer detection in the future.

## Perspective

A growing number of circulating miRNAs were found to have potential to act as diagnostic or prognostic biomarkers for various types of cancer patients, especially for long-term, slow progress solid tumors which are hard to detect at early stage. However, the difference between criteria of scientific research and clinical application is quite obvious. This gap makes it important to carefully examine for any potential miRNA biomarkers. An appropriate applicable biomarker for specific cancer should not only be significantly differentially expressed, but also be capable of defining the correlation with the outcome of patients.

The limited sample size was another unavoidable obstacle. In practical clinical application, the level of circulating biomarkers would be under the influence of multiple individual classifications, including age, gender, ethnic, lifestyle, history of diseases, and so on. Although many investigators validated that the miRNAs they focused were not affected by individual characteristics, the proportion may be limited, and larger sample size cohort studies were still needed for further assessment. Detection results could also be affected by measurement principle, method, instrument, and the operation of technicians. So expanding the sample size could be a critical process to ensure the accuracy for cancer diagnosis. In addition, the absolute quantitative detection method may be promising as well as a basic level of the circulating biomarker in healthy controls.

Differentially expressed circulating miRNAs may also play other roles for patients suffering from different cancers except for functioning as biomarkers. Some of them may be just results or by-products of diseases, and the others participated in the occurrence and development of tumors directly or indirectly. There are few studies to reveal the association between the levels of aberrant expressions of miRNAs and therapeutic options so far. However, those miRNAs that participated in tumor pathology may reflect and in turn change the cellular transcriptome via complex regulatory network. Exogenous overexpression or inhibition of those functional miRNAs would probably rescue the pathological development and do favor to the treatment and improvement. Therefore, the circulating miRNA biomarkers could further serve as valuable research targets and candidate small molecule drugs for clinical treatment.

So far, multiple independent validation studies are still demanded for clinical application. The combination of miRNAs with other biomarkers and precise selection of their origin could contribute to further researches. Obtaining the comprehensive view of miRNAs, identified and unidentified, as well as the lncRNAs, circular-RNAs, and other ncRNAs is still on the way. With clearer regulatory guidance, a more precise approach could be expected to provide a promising detection to improve treatment outcomes.

## Conclusion

In conclusion, circulating miRNAs exhibited promising potential to serve as effective non-invasive cancer biomarkers for clinical application. They may be valuable in various aspects including cancer screening in the early stage, subtype classification and drug sensitivity prediction for treatment strategy selection, and screening the chemo- or radio-resistance of tumors to prognosis the outcomes and recurrences. Larger scale studies are expected to further promote the sensitivity, specificity, and applicability of potential circulating miRNA biomarkers in the future.

## Abbreviations

ADC: Adenocarcinoma
AFP: Alpha-fetoprotein
BMI: Body mass index
CA: Carbohydrate antigen
ccRCC: Clear-cell renal cell cancer
CEA: Carcinoembryonic antigen
CRC: Colorectal cancer
DFS: Disease-free survival
ER: Estrogen receptor
ESCC: Esophageal squamous cell carcinoma
miRNA: MicroRNA
NSCLC: Non-small cell lung cancers
OS: Overall survival
PCR: Polymerase chain reaction
PDAC: Pancreatic ductal adenocarcinoma
PR: Progesterone receptor
PSA: Prostate-specific antigen
SCC: Squamous cell carcinoma
TNBC: Triple-negative breast cancers

## References

[1] Lee RC, Feinbaum RL, Ambros V (1993). The C. elegans heterochronic gene lin-4 encodes small RNAs with antisense complementarity to lin-14. Cell.

[2] Bracken CP, Scott HS, Goodall GJ (2016). A network-biology perspective of microRNA function and dysfunction in cancer. Nat Rev Genet.

[3] Baek D, Villen J, Shin C, Camargo FD, Gygi SP, Bartel DP (2008). The impact of microRNAs on protein output. Nature.

[4] Bushati N, Cohen SM (2007). microRNA functions. Annu Rev Cell Dev Biol.

[5] Zhu Y, Xiong K, Shi J, Cui Q, Xue L (2017). A potential role of microRNAs in protein accumulation in cellular senescence analyzed by bioinformatics. PLoS One.

[6] Lin S, Gregory RI (2015). MicroRNA biogenesis pathways in cancer. Nat Rev Cancer.

[7] Calin GA, Croce CM (2006). MicroRNA signatures in human cancers. Nat Rev Cancer.

[8] Rosenfeld N, Aharonov R, Meiri E, Rosenwald S, Spector Y, Zepeniuk M (2008). MicroRNAs accurately identify cancer tissue origin. Nat Biotechnol.

[9] Berezikov E, Chung WJ, Willis J, Cuppen E, Lai EC (2007). Mammalian mirtron genes. Mol Cell.

[10] Flynt AS, Greimann JC, Chung WJ, Lima CD, Lai EC (2010). MicroRNA biogenesis via splicing and exosome-mediated trimming in Drosophila. Mol Cell.

[11] Westholm JO, Lai EC (2011). Mirtrons: microRNA biogenesis via splicing. Biochimie.

[12] Valadi H, Ekström K, Bossios A, Sjöstrand M, Lee JJ, Lötvall JO (2007). Exosome-mediated transfer of mRNAs and microRNAs is a novel mechanism of genetic exchange between cells. Nat Cell Biol.

[13] Mitchell PS, Parkin RK, Kroh EM, Fritz BR, Wyman SK, Pogosova-Agadjanyan EL (2008). Circulating microRNAs as stable blood-based markers for cancer detection. Proc Natl Acad Sci U S A.

[14] Shen J, Stass SA, Jiang F (2013). MicroRNAs as potential biomarkers in human solid tumors. Cancer Lett.

[15] Lindner K, Haier J, Wang Z, Watson DI, Hussey DJ, Hummel R (2015). Circulating microRNAs: emerging biomarkers for diagnosis and prognosis in patients with gastrointestinal cancers. Clin Sci (Lond).

[16] Vanni I, Alama A, Grossi F, Dal Bello MG, Coco S (2017). Exosomes: a new horizon in lung cancer. Drug Discov Today.

[17] Zhang H, Mao F, Shen T, Luo Q, Ding Z, Qian L, Huang J (2017). Plasma miR-145, miR-20a, miR-21 and miR-223 as novel biomarkers for screening early-stage non-small cell lung cancer. Oncol Lett.

[18] Geng Q, Fan T, Zhang B, Wang W, Xu Y, Hu H (2014). Five microRNAs in plasma as novel biomarkers for screening of early-stage non-small cell lung cancer. Respir Res.

[19] Arab A, Karimipoor M, Irani S, Kiani A, Zeinali S, Tafsiri E, Sheikhy K (2017). Potential circulating miRNA signature for early detection of NSCLC. Cancer Genet.

[20] Zhu W, Zhou K, Zha Y, Chen D, He J, Ma H (2016). Diagnostic value of serum miR-182, miR-183, miR-210, and miR-126 levels in patients with early-stage non-small cell lung cancer. PLoS One.

[21] Powrózek T, Kuźnar-Kamińska B, Dziedzic M, Mlak R, Batura-Gabryel H, Sagan D (2017). The diagnostic role of plasma circulating precursors of miRNA-944 and miRNA-3662 for non-small cell lung cancer detection. Pathol Res Pract.

[22] Wang J, Yan F, Zhao Q, Zhan F, Wang R, Wang L (2017). Circulating exosomal miR-125a-3p as a novel biomarker for early-stage colon cancer. Sci Rep.

[23] Vychytilova-Faltejskova P, Radova L, Sachlova M, Kosarova Z, Slaba K, Fabian P (2016). Serum-based microRNA signatures in early diagnosis and prognosis prediction of colon cancer. Carcinogenesis.

[24] Hamam R, Ali AM, Alsaleh KA, Kassem M, Alfayez M, Aldahmash A, Alajez NM (2016). microRNA expression profiling on individual breast cancer patients identifies novel panel of circulating microRNA for early detection. Sci Rep.

[25] Xing W, Zeng F (2017). A novel serum microRNA-based identification and classification biomarker of human glioma. Tumour Biol.

[26] Blandin Knight S, Grosbie PA, Balata H, Chudziak J, Hussell T, Dive C (2017). Progress and prospects of early detection in lung cancer. Open Biol.

[27] Hon JD, Singh B, Sahin A, Du G, Wang J, Wang VY (2016). Breast cancer molecular subtypes: from TNBC to QNBC. Am J Cancer Res.

[28] Rahim B, O'Regan R (2017). AR signaling in breast cancer. Cancers (Basel).

[29] Sromek M, Glogowski M, Chechlinska M, Kulinczak M, Szafron L, Zakrzewska K (2017). Changes in plasma miR-9, miR-16, miR-205 and miR-486 levels after non-small cell lung cancer resection. Cell Oncol (Dordr).

[30] Jin X, Chen Y, Chen H, Fei S, Chen D, Cai X (2017). Evaluation of tumor-derived exosomal miRNA as potential diagnostic biomarkers for early-stage non-small cell lung cancer using next-generation sequencing. Clin Cancer Res.

[31] Powrózek T, Krawczyk P, Kowalski DM, Winiarczyk K, Olszyna-Serementa M, Milanowski J (2015). Plasma circulating microRNA-944 and microRNA-3662 as potential histologic type-specific early lung cancer biomarkers. Transl Res.

[32] Shin VY, Siu JM, Cheuk I, Ng EK, Kwong A (2015). Circulating cell-free miRNAs as biomarker for triple-negative breast cancer. Br J Cancer.

[33] Eichelser C, Stückrath I, Müller V, Milde-Langosch K, Wikman H, Pantel K, Schwarzenbach H (2017). Increased serum levels of circulating exosomal microRNA-373 in receptor-negative breast cancer patients. Oncotarget.

[34] Perge P, Butz H, Pezzani R, Bancos I, Nagy Z, Paloczi K (2017). Evaluation and diagnostic potential of circulating extracellular vesicle-associated microRNAs in adrenocortical tumors. Sci Rep.

[35] Samsonov R, Burdakov V, Shtam T, Radzhabovа Z, Vasilyev D, Tsyrlina E (2016). Plasma exosomal miR-21 and miR-181a differentiates follicular from papillary thyroid cancer. Tumour Biol.

[36] Pang PC, Shi XY, Huang WL, Sun K (2016). miR-497 as a potential serum biomarker for the diagnosis and prognosis of osteosarcoma. Eur Rev Med Pharmacol Sci.

[37] Wang C, Li Q, Liu F, Chen X, Nesa EU, Guan S (2016). Downregulation of microRNA-497 is associated with upregulation of synuclein gamma in patients with osteosarcoma. Exp Ther Med.

[38] Liu Q, Wang H, Singh A, Shou F (2016). Expression and function of microRNA-497 in human osteosarcoma. Mol Med Rep.

[39] Shao XJ, Miao MH, Xue J, Xue J, Ji XQ, Zhu H (2015). The down-regulation of microRNA-497 contributes to cell growth and cisplatin resistance through PI3K/Akt pathway in osteosarcoma. Cell Physiol Biochem.

[40] Ruan WD, Wang P, Feng S, Xue Y, Zhang B (2016). MicroRNA-497 inhibits cell proliferation, migration, and invasion by targeting AMOT in human osteosarcoma cells. Onco Targets Ther.

[41] Ge L, Zheng B, Li M, Niu L, Li Z (2016). MicroRNA-497 suppresses osteosarcoma tumor growth in vitro and in vivo. Oncol Lett.

[42] Gui ZL (2017). MicroRNA-497 suppress osteosarcoma by targeting MAPK/Erk pathway. Bratisl Lek Listy.

[43] Fang Z (2016). Circulating miR-205: a promising biomarker for the detection and prognosis evaluation of bladder cancer. Tumour Biol.

[44] Gong ZL, Wu TL, Zhao GC, Lin ZX, Xu HG (2016). Decreased expression of microRNA-148a predicts poor prognosis in ovarian cancer and associates with tumor growth and metastasis. Biomed Pharmacother.

[45] Ren GB, Wang L, Zhang FH, Meng XR, Mao ZP (2016). Study on the relationship between miR-520g and the development of breast cancer. Eur Rev Med Pharmacol Sci.

[46] Yuan R, Wang G, Xu Z, Zhao H, Chen H, Han Y (2016). Up-regulated circulating miR-106a by DNA methylation promised a potential diagnostic and prognostic marker for gastric cancer. Anti Cancer Agents Med Chem.

[47] Tsai MM, Wang CS, Tsai CY, Huang CG, Lee KF, Huang HW (2016). Circulating microRNA-196a/b are novel biomarkers associated with metastatic gastric cancer. Eur J Cancer.

[48] Papaconstantinou IG, Manta A, Gazouli M, Lyberopoulou A, Lykoudis PM, Polymeneas G, Voros D (2013). Expression of microRNAs in patients with pancreatic cancer and its prognostic significance. Pancreas.

[49] Greither T, Grochola LF, Udelnow A, Lautenschlager C, Wurl P, Taubert H (2010). Elevated expression of microRNAs 155, 203, 210 and 222 in pancreatic tumors is associated with poorer survival. Int J Cancer.

[50] Wang J, Chen J, Chang P, LeBlanc A, Li D, Abbruzzesse JL (2009). MicroRNAs in plasma of pancreatic ductal adenocarcinoma patients as novel blood-based biomarkers of disease. Cancer Prev Res (Phila).

[51] Mikamori M, Yamada D, Eguchi H, Hasegawa S, Kishimoto T, Tomimaru Y (2017). MicroRNA-155 controls exosome synthesis and promotes gemcitabine resistance in pancreatic ductal adenocarcinoma. Sci Rep.

[52] Hu J, Cai G, Xu Y, Cai S (2016). The plasma microRNA miR-1914* and -1915 suppresses chemoresistant in colorectal cancer patients by down-regulating NFIX. Curr Mol Med.

[53] Yuwen DL, Sheng BB, Liu J, Wenyu W, Shu YQ (2017). MiR-146a-5p level in serum exosomes predicts therapeutic effect of cisplatin in non-small cell lung cancer. Eur Rev Med Pharmacol Sci.

[54] Wei F, Ma C, Zhou T, Dong X, Luo Q, Geng L (2017). Exosomes derived from gemcitabine-resistant cells transfer malignant phenotypic traits via delivery of miRNA-222-3p. Mol Cancer.

[55] Liu Q, Yu Z, Yuan S, Xie W, Li C, Hu Z (2017). Circulating exosomal microRNAs as prognostic biomarkers for non-small-cell lung cancer. Oncotarget.

[56] Dejima H, Iinuma H, Kanaoka R, Matsutani N, Kawamura M (2017). Exosomal microRNA in plasma as a non-invasive biomarker for the recurrence of non-small cell lung cancer. Oncol Lett.

[57] Tsukamoto M, Iinuma H, Yagi T, Matsuda K, Hashiguchi Y (2017). Circulating exosomal microRNA-21 as a biomarker in each tumor stage of colorectal cancer. Oncology.

[58] Manier S, Liu CJ, Avet-Loiseau H, Park J, Shi J, Campigotto F (2017). Prognostic role of circulating exosomal miRNAs in multiple myeloma. Blood.

[59] Liu C, Eng C, Shen J, Lu Y, Takata Y, Mehdizadeh A (2016). Serum exosomal miR-4772-3p is a predictor of tumor recurrence in stage II and III colon cancer. Oncotarget.

[60] Gu Y, Zhang M, Peng F, Fang L, Zhang Y, Liang H (2015). The BRCA1/2-directed miRNA signature predicts a good prognosis in ovarian cancer patients with wild-type BRCA1/2. Oncotarget.

[61] Tang H, Liu Q, Liu X, Ye F, Xie X, Xie X, Wu M (2015). Plasma miR-185 as a predictive biomarker for prognosis of malignant glioma. J Cancer Res Ther.

[62] Ugrinska A, Bombardieri E, Stokkel MP, Crippa F, Pauwels EK (2002). Circulating tumor markers and nuclear medicine imaging modalities: breast, prostate and ovarian cancer. Q J Nucl Med.

[63] Wang Y, Yan J, Wang L (2014). The diagnostic value of serum carcino-embryonic antigen, alpha fetoprotein and carbohydrate antigen 19-9 for colorectal cancer. J Cancer Res Ther.

[64] Baltzer PAT, Kapetas P, Marino MA, Clauser P (2017). New diagnostic tools for breast cancer. Memo.

[65] Modiri A, Goudreau S, Rahimi A, Kiasaleh K (2017). Review of breast screening: toward clinical realization of microwave imaging. Med Phys.

[66] Issa IA, Noureddine M (2017). Colorectal cancer screening: an updated review of the available options. World J Gastroenterol.

[67] Sadi MV (2017). PSA screening for prostate cancer. Rev Assoc Med Bras (1992).

[68] He X, Ping J, Wen D (2017). MicroRNA-186 regulates the invasion and metastasis of bladder cancer via vascular endothelial growth factor C. Exp Ther Med.

[69] Geva GA, Gielchinsky I, Aviv N, Max KEA, Gofrit ON, Gur-Wahnon D, Ben-Dov IZ (2017). Urine cell-free microRNA as biomarkers for transitional cell carcinoma. BMC Res Notes.

[70] Fedorko M, Juracek J, Stanik M, Svoboda M, Poprach A, Buchler T (2017). Detection of let-7 miRNAs in urine supernatant as potential diagnostic approach in non-metastatic clear-cell renal cell carcinoma. Biochem Med (Zagreb).

[71] Butz H, Nofech-Mozes R, Ding Q, Khella HWZ, Szabó PM, Jewett M (2016). Exosomal microRNAs are diagnostic biomarkers and can mediate cell-cell communication in renal cell carcinoma. Eur Urol Focus.

[72] Erbes T, Hirschfeld M, Rucker G, Jaeger M, Boas J, Iborra S (2015). Feasibility of urinary microRNA detection in breast cancer patients and its potential as an innovative non-invasive biomarker. BMC Cancer.

[73] Debernardi S, Massat NJ, Radon TP, Sangaralingam A, Banissi A, Ennis DP (2015). Noninvasive urinary miRNA biomarkers for early detection of pancreatic adenocarcinoma. Am J Cancer Res.

[74] Zhou Y, Tian L, Wang X, Ye L, Zhao G, Yu M, et al. MicroRNA-195 inhibits non-small cell lung cancer cell proliferation, migration and invasion by targeting MYB. Cancer Lett 2014; 347: 65–74. 10.1016/j.canlet.2014.01.019 24486218

[75] Wang X (2014). MiR-195 inhibits the growth and metastasis of NSCLC cells by targeting IGF1R. Tumour Biol.

[76] Guo H, Li W, Zheng T, Liu Z (2014). MiR-195 targets HDGF to inhibit proliferation and invasion of NSCLC cells. Tumour Biol.

[77] Liu B, Qu J, Xu F, Guo Y, Wang Y, Yu H, Qian B (2015). MiR-195 suppresses non-small cell lung cancer by targeting CHEK1. Oncotarget.

[78] Su K, Zhang T, Wang Y, Hao G (2016). Diagnostic and prognostic value of plasma microRNA-195 in patients with non-small cell lung cancer. World J Surg Oncol.

[79] Hua Y, Chen H, Wang L, Wang F, Wang P, Ning Z (2017). Low serum miR-373 predicts poor prognosis in patients with pancreatic cancer. Cancer Biomark.

[80] Murali Manohar K, Sasikala M, Kvsrr Y, Sunil V, Talukdar R, Murthy H (2017). Plasma microRNA192 in combination with serum CA19-9 as non-invasive prognostic biomarker in periampullary carcinoma. Tumour Biol.

[81] Zeng Z, Chen X, Zhu D, Luo Z, Yang M (2017). Low expression of circulating microRNA-34c is associated with poor prognosis in triple-negative breast cancer. Yonsei Med J.

[82] Huang Z, Zhang L, Zhu D, Shan X, Zhou X, Qi LW (2017). A novel serum microRNA signature to screen esophageal squamous cell carcinoma. Cancer Med.

[83] Xu W, Zhou Y, Xu G, Geng B, Cui Q (2017). Transcriptome analysis reveals non-identical microRNA profiles between arterial and venous plasma. Oncotarget.

[84] Zhou X, Wen W, Shan X, Zhu W, Xu J, Guo R (2017). A six-microRNA panel in plasma was identified as a potential biomarker for lung adenocarcinoma diagnosis. Oncotarget.

[85] Monzo M, Santasusagna S, Moreno I, Martinez F, Hernández R, Muñoz C (2017). Exosomal microRNAs isolated from plasma of mesenteric veins linked to liver metastases in resected patients with colon cancer. Oncotarget.

[86] Tian F, Shen Y, Chen Z, Li R, Ge Q (2017). No significant difference between plasma miRNAs and plasma-derived exosomal miRNAs from healthy people. Biomed Res Int.

[87] Huang Z, Zhu D, Wu L, He M, Zhou X, Zhang L (2017). Six serum-based miRNAs as potential diagnostic biomarkers for gastric cancer. Cancer Epidemiol Biomark Prev.

[88] Heegaard NH, Schetter AJ, Welsh JA, Yoneda M, Bowman ED, Harris CC (2012). Circulating micro-RNA expression profiles in early stage nonsmall cell lung cancer. Int J Cancer.

[89] Chen H, Liu H, Zou H, Chen R, Dou Y, Sheng S (2016). Evaluation of plasma miR-21 and miR-152 as diagnostic biomarkers for common types of human cancers. J Cancer.

[90] Eichelser C, Flesch-Janys D, Chang-Claude J, Pantel K, Schwarzenbach H (2013). Deregulated serum concentrations of circulating cell-free microRNAs miR-17, miR-34a, miR-155, and miR-373 in human breast cancer development and progression. Clin Chem.

[91] Sochor M, Basova P, Pesta M, Dusilkova N, Bartos J, Burda P (2014). Oncogenic microRNAs: miR-155, miR-19a, miR-181b, and miR-24 enable monitoring of early breast cancer in serum. BMC Cancer.

[92] Madhavan D, Peng C, Wallwiener M, Zucknick M, Nees J, Schott S (2016). Circulating miRNAs with prognostic value in metastatic breast cancer and for early detection of metastasis. Carcinogenesis.

[93] Wang W, Qu A, Liu W, Liu Y, Zheng G, Du L, et al. Circulating miR-210 as a diagnostic and prognostic biomarker for colorectal cancer. Eur J Cancer Care (Engl). 2017;26:e12448 10.1111/ecc.12448 26898324

[94] Ulivi P, Canale M, Passardi A, Marisi G, Valgiusti M, Frassineti GL, et al. Circulating plasma levels of miR-20b, miR-29b and miR-155 as predictors of bevacizumab efficacy in patients with metastatic colorectal cancer. Int J Mol Sci. 2018;19:307 10.3390/ijms19010307 PMC5796251 29361687

[95] Montagnana M, Benati M, Danese E, Minicozzi AM, Paviati E, Gusella M (2016). Plasma expression levels of circulating miR-21 are not useful for diagnosing and monitoring colorectal cancer. Clin Lab.

[96] Sierzega M, Kaczor M, Kolodziejczyk P, Kulig J, Sanak M, Richter P (2017). Evaluation of serum microRNA biomarkers for gastric cancer based on blood and tissue pools profiling: the importance of miR-21 and miR-331. Br J Cancer.

[97] Zheng Y, Cui L, Sun W, Zhou H, Yuan X, Huo M (2011). MicroRNA-21 is a new marker of circulating tumor cells in gastric cancer patients. Cancer Biomark.

[98] Sohn W, Kim J, Kang SH, Yang SR, Cho JY, Cho HC, Shim SG, Paik YH (2015). Serum exosomal microRNAs as novel biomarkers for hepatocellular carcinoma. Exp Mol Med.

[99] Zhang Y, Zhang D, Wang F, Xu D, Guo Y, Cui W (2015). Serum miRNAs panel (miR-16-2*, miR-195, miR-2861, miR-497) as novel non-invasive biomarkers for detection of cervical cancer. Sci Rep.

[100] Lian F, Cui Y, Zhou C, Gao K, Wu L (2015). Identification of a plasma four-microRNA panel as potential noninvasive biomarker for osteosarcoma. PLoS One.

[101] Summerer I, Unger K, Braselmann H, Schuettrumpf L, Maihoefer C, Baumeister P (2015). Circulating microRNAs as prognostic therapy biomarkers in head and neck cancer patients. Br J Cancer.

[102] Zhou X, Wen W, Zhu J, Huang Z, Zhang L, Zhang H (2017). A six-microRNA signature in plasma was identified as a potential biomarker in diagnosis of esophageal squamous cell carcinoma. Oncotarget.

[103] Osip'yants AI, Knyazev EN, Galatenko AV, Nyushko KM, Galatenko VV, Shkurnikov MY, Alekseev BY (2017). Changes in the level of circulating hsa-miR-297 and hsa-miR-19b-3p miRNA are associated with generalization of prostate cancer. Bull Exp Biol Med.

